# Revised Estimates for the Number of Human and Bacteria Cells in the Body

**DOI:** 10.1371/journal.pbio.1002533

**Published:** 2016-08-19

**Authors:** Ron Sender, Shai Fuchs, Ron Milo

**Affiliations:** 1 Department of Plant and Environmental Sciences, Weizmann Institute of Science, Rehovot, Israel; 2 Department of Molecular Genetics, Weizmann Institute of Science, Rehovot, Israel

## Abstract

Reported values in the literature on the number of cells in the body differ by orders of magnitude and are very seldom supported by any measurements or calculations. Here, we integrate the most up-to-date information on the number of human and bacterial cells in the body. We estimate the total number of bacteria in the 70 kg "reference man" to be 3.8·10^13^. For human cells, we identify the dominant role of the hematopoietic lineage to the total count (≈90%) and revise past estimates to 3.0·10^13^ human cells. Our analysis also updates the widely-cited 10:1 ratio, showing that the number of bacteria in the body is actually of the same order as the number of human cells, and their total mass is about 0.2 kg.

## Abstract 2

Thoroughly revised estimates show that the typical adult human body consists of about 30 trillion human cells and about 38 trillion bacteria.

## Introduction

How many cells are there in the human body? Beyond order of magnitude statements that give no primary reference or uncertainty estimates, very few detailed estimates have been performed (the one exception [1] is discussed below). Similarly, the ubiquitous statements regarding 10^14^–10^15^ bacteria residing in our body trace back to an old back-of-the-envelope calculation [2–4].

The aim of this study is to critically revisit former estimates for the number of human and bacterial cells in the human body. We give up-to-date detailed estimates where the calculation logic and sources are fully documented and uncertainty ranges are derived. By updating the cell counts in the body, we also revisit the 10:1 value that has been so thoroughly repeated as to achieve the status of an established common knowledge fact [4]. This ratio was criticized recently in a letter to the journal *Microbe* [5], but an alternative detailed estimate that will give concrete values and estimate the uncertainty range is needed. Here, we provide an account of the methodologies employed hitherto for cell count and revise past estimates. Doing so, we repeat and reflect on the assumptions in previous back-of-the-envelope calculations, also known as Fermi problems. We find such estimates as effective sanity checks and a way to improve our quantitative understanding in biology.

A major part of the available literature used in the derivation of human cell numbers was based on cohorts of exclusively or mostly men, and as we use these sources, our analysis starts with adult men. As discussed below, relatively modest quantitative differences apply for women due to changes in characteristic body mass, blood volume, and the genital microbiota. For our analysis, we used the definition of the standard reference man as given in the literature [6] as: "Reference Man is defined as being between 20–30 years of age, weighing 70 kg, is 170 cm in height.” Our analysis revisits the estimates for the number of microbial cells, human cells, and their ratio in the body of such a standard man.

We begin our analysis by revisiting the number of bacteria through surveying earlier sources, comparing counts in different body organs and finally focusing on the content of the colon. We then estimate the total number of human cells in the body, comparing calculations using a "representative" cell size to aggregation by cell type. We then contrast the cell number distribution by tissue type to the mass distribution. In closing, we revisit the ratio of bacterial to human cells and evaluate the effect of gender, age, and obesity.

## Results

### Origin of Prevalent Claims in the Literature on the Number of Bacterial Cells in Humans

Microbes are found throughout the human body, mainly on the external and internal surfaces, including the gastrointestinal tract, skin, saliva, oral mucosa, and conjunctiva. Bacteria overwhelmingly outnumber eukaryotes and archaea in the human microbiome by 2–3 orders of magnitude [7,8]. We therefore sometimes operationally refer to the microbial cells in the human body as bacteria. The diversity in locations where microbes reside in the body makes estimating their overall number daunting. Yet, once their quantitative distribution shows the dominance of the colon as discussed below, the problem becomes much simpler. The vast majority of the bacteria reside in the colon, with previous estimates of about 10^14^ bacteria [2], followed by the skin, which is estimated to harbor ~10^12^ bacteria [9].

As we showed recently [4], all papers regarding the number of bacteria in the human gastrointestinal tract that gave reference to the value stated could be traced to a single back-of-the-envelope estimate [3]. That order of magnitude estimate was made by assuming 10^11^ bacteria per gram of gut content and multiplying it by 1 liter (or about 1 kg) of alimentary tract capacity. To get a revised estimate for the overall number of bacteria in the human body, we first discuss the quantitative distribution of bacteria in the human body. After showing the dominance of gut bacteria, we revisit estimates of the total number of bacteria in the human body.

### Distribution of Bacteria in Different Human Organs

Table 1 shows typical order of magnitude estimates for the number of bacteria that reside in different organs in the human body. The estimates are based on multiplying measured concentrations of bacteria by the volume of each organ [9,10]. Values are rounded up to give an order of magnitude upper bound.

**Table 1:** Bounds for bacteria number in different organs, derived from bacterial concentrations and volume.

Location	Typical concentration of bacteria ^(1)^(number/mL content)	Volume (mL)	Order of magnitude bound for bacteria number
Colon (large intestine)	10^11^	400 ^(2)^	10^14^
Dental plaque	10^11^	<10	10^12^
Ileum (lower small intestine)	10^8^	400 ^(5)^	10^11^
Saliva	10^9^	<100	10^11^
Skin	<10^11^ per m^2^ ^(3)^	1.8 m^2^ ^(4)^	10^11^
Stomach	10^3^–10^4^	250 ^(5)^–900 ^(6)^	10^7^
Duodenum and Jejunum (upper small intestine)	10^3^–10^4^	400 ^(5)^	10^7^

^(1)^ Except for skin, concentrations are according to [9]. For the skin, we used bacterial areal density and total skin surface to reach an upper bound.

^(2)^ See derivation in section below.

^(3)^ Skin surface bacteria density is taken from [11].

^(4)^ Skin area calculated as inferred from standard formula by DuBois for the body surface area [12].

^(5)^ Volume of the organs of the gastrointestinal tract is derived from weights taken from [13] by assuming content density of 1.04 g/mL [6].

^(6)^ Higher value is given in [14].

Although the bacterial concentrations in the saliva and dental plaque are high, because of their small volume the overall numbers of bacteria in the mouth are less than 1% of the colon bacteria number. The concentration of bacteria in the stomach and the upper 2/3 of the small intestine (duodenum and jejunum) is only 10^3^–10^4^ bacteria/mL, owing to the relatively low pH of the stomach and the fast flow of the content through the stomach and the small intestine [10]. Table 1 reveals that the bacterial content of the colon exceeds all other organs by at least two orders of magnitude. Importantly, within the alimentary tract, the colon is the only substantial contributor to the total bacterial population, while the stomach and small intestine make negligible contributions.

### Revisiting the Original Back-of-the-Envelope Estimate for the Number of Bacteria in the Colon

The primary source for the often cited value of ~10^14^ bacteria in the body dates back to the 1970s [3] and only consists of a sentence-long “derivation,” which assumes the volume of the alimentary tract to be 1 liter, and multiplies this volume by the number density of bacteria, known to be about 10^11^ bacteria per gram of wet content. Such estimates are often very illuminating, yet it is useful to revisit them as more empirical data accumulates. This pioneering estimate of 10^14^ bacteria in the intestine is based on assuming a constant bacterial density over the 1 liter of alimentary tract volume (converting from volume to mass via a density of 1 g/mL). Yet, the parts of the alimentary tract proximal to the colon contain a negligible number of bacteria in comparison to the colon content, as can be appreciated from Table 1. We thus conclude that the relevant volume for the high bacteria density of 10^11^ bacteria/g is only that of the colon. As discussed in Box 1, we integrated data sources on the volume of the colon to arrive at 0.4 L.

**Box 1. The Volume of the Human Colon Content**

This is a critical parameter in our calculation. We used a value of 0.4 L based on the following studies (see also S1 Data, tab ColonContent). The volume of the colon content of the reference adult man was previously estimated as 340 mL (355 g at density of 1.04 g/mL [6]), based on various indirect methods including flow measurements, barium meal X-ray measurements and postmortem examination [13]. A recent study [15] gives more detailed data about the volume of undisturbed colon that was gathered by MRI scans. The authors report a height-standardized colonic inner volume for males of 97 ± 24 mL/m^3^ (where the best fit was found when dividing the colonic volume by the cube of the height). Taking a height of 1.70 m for the reference man [6], we arrive at a colon volume of 480 ± 120 mL (where unless noted otherwise ± refers to the standard deviation [SD]). This volume includes an unreported volume of gas and did not include the rectum. Most recently, studies analyzing MRI images of the colon provided the most detailed and complete data. The inner colon volume in that cohort was 760 mL in total [16,17]. This cohort was, however, significantly taller than the reference man. Normalizing for height, we arrive at 600 mL total volume for a standard man. In order to deduct the volume occupied by gas, stool fraction in this report was estimated at ≈70% of colon volume leading to 430 mL of standardized wet colon content. Therefore, this most reliable analysis together with earlier studies support an average value of about 0.4 L.

We can sanity-check this volume estimate by looking at the volume of stool that flows through the colon. An adult human is reported to produce on average 100–200 grams of wet stool per day [18]. The colonic transit time is negatively correlated with the daily fecal output, and its normal values are about 25–40 hours [18,19]. By multiplying the daily output and the colon transit time, we thus get a volume estimate of 150–250 mL, which is somewhat lower than but consistent with the values above, given the uncertainties and very crude estimate that did not account for water in the colon that is absorbed before defecation. To summarize, the volume of colon content as evaluated by recent analyses of MRI images is in keeping with previous estimates and fecal transit dynamics. Values for a reference adult man averaged 0.4 L (standard error of the mean [SEM] 17%, coefficient of variation [CV] 25%), which will be used in calculations below. Following a typical meal, the volume changes by about 10% [15], while each defecation event reduces the content by a quarter to a third [18].

### The Total Number of Bacteria in the Body

We are now able to repeat the original calculation for the number of bacteria in the colon [3]. Given 0.9·10^11^ bacteria/g wet stool as derived in Box 2 and 0.4 L of colon, we find 3.8·10^13^ bacteria in the colon with a standard error uncertainty of 25% and a variation of 52% SD over a population of 70 kg males. Considering that the contribution to the total number of bacteria from other organs is at most 10^12^, we use 3.8·10^13^ as our estimate for the number of bacteria across the whole body of the "reference man."

**Box 2. Concentration of Bacteria in the Colon**

The most widely used approach for measuring the bacterial cell density in the colon is by examining bacteria content in stool samples. This assumes that stool samples give adequate representation of colon content. We return to this assumption in the discussion. The first such experiments date back to the 1960s and 1970s [20,21]. In those early studies, counting was based on direct microscopic clump counts from diluted stool samples. Later experiments [22,23] used DAPI nucleic acid staining and fluorescent in situ hybridization [FISH] to bacterial 16S RNA. Values are usually reported as bacteria per gram of dry stool. For our calculation, we are interested in the bacteria content for the wet rather than dry content of the colon. To move from ^bacteria^/_g dry stool_ to ^bacteria^/_g wet stool_ we use the fraction of dry matter as reported in each article. Table 2 reports the values we extracted from 14 studies in the literature and translated them to a common basis enabling comparison.

**Table 2:** Values of bacteria density in stool as reported in several past articles.

Article		bac. #/g dry stool (x10^11^)	dry matter as % of stool	bac. #/g wet stool (x10^11^)	CV(%)
Author	Year				
Houte & Gibbons	1966	-	-	**3.2**	53%
Moore & Holdeman	1974	**5**	**22%**	**1.1**	78%
Holdeman, Good & Moore	1976	**4.1**	**31%**	*1*.*3*	66%
Stephen & Cummings	1980	**4**	29%^(1)^	*1*.*2*	25%
Langendijk et al.	1995	-	-	**2.7**	26%
Franks et al.	1998	**2.9**	-	*0*.*74*^(2)^	39%
Simmering & Kleessen	1999	**4.8**	-	*1*.*3*^(2)^	44%
Tannock et al.	2000	-	-	**0.95**	40%
Harmsen, Raangs, He, Degener & Welling	2002	**2.1**	*30%*	**0.62**	38%
Zoetendal et al	2002	**2.9**	-	*0*.*77*^(2)^	24%
Zhong et al.	2004	**1.5**	**23%**	*0*.*35*	73%
Thiel & Blaut	2005	**3.5**	*25%*	**0.87**	53%
He et al.	2008	**1.5**	-	*0*.*39*^(2)^	43%
Uyeno, Sekiguchi & Kamagata	2008	-	-	**0.44**	34%
**Mean**		-	**27% ± 2%**	**0.92 ± 19%**	**46%**

Full references are provided in Table A in S1 Appendix. Mean bacteria number is calculated using the geometric mean to give robustness towards outlier values. Values quoted directly from the articles are written in bold, values derived by us are written in italic. Values reported with more than two significant digits are rounded to two significant digits as the uncertainty makes such overspecification nonsensible. ± standard error of the mean.

^(1)^ Value for [21] derived from their Table 1.

^(2)^ From derivation, assuming the averaged dry matter fraction of 27%.

From the measurements collected in Table 2, we calculated the representative bacteria concentration in the colon by two methods, yielding very close values: the geometric mean is 0.92·10^11^ (SEM 19%) bacteria per gram of wet stool, while the median of the values is 0.91·10^11^ (SEM 19% by bootstrapping, see methods in S1 Appendix). The variation across the population, given by the average CV, is 46%.

We note that the uncertainty estimate value takes into account known variation in the colon volume, bacteria density, etc., but cannot account for unquantified systematic biases. One prominent such bias is the knowledge gap on differences between the actual bacteria density in the colon, with all its spatial heterogeneity, and the measurements of concentration in feces, which serve as the proxy for estimating bacteria number.

What is the total mass of bacteria in the body? From the total colon content of about 0.4 kg and a bacteria mass fraction of about one-half [21,24], we get a contribution of about 0.2 kg (wet weight) from bacteria to the overall mass of the colon content. Given the dominance of bacteria in the colon over all other microbiota populations in the body, we conclude that there is about 0.2 kg of bacteria in the body overall. Given the water content of bacteria, the total dry weight of bacteria in the body is about 50–100g. This value is consistent with a parallel alternative estimate for the total mass of bacteria that multiplies the average mass of a gut bacterium of about 5 pg (wet weight, corresponding to a dry weight of 1–2 pg, see S1 Appendix) with the updated total number of bacteria. We note that this empirically observed average gut bacterium is several times bigger than the conveniently chosen “standard” 1 μm^3^ volume and 1 pg wet mass bacterium often referred to in textbooks. The total bacteria mass we find represents about 0.3% of the overall body weight, significantly updating previous statements that 1%–3% of the body mass is composed of bacteria or that a normal human hosts 1–3 kg of bacteria [25].

### The Number of Human Cells in a “Standard” Adult Male

Many literature sources make general statements on the number of cells in the human body ranging between 10^12^ to 10^14^ cells [26,27]. An order of magnitude back-of-the-envelope argument behind such values is shown in Box 3.

**Box 3. Order of Magnitude, Naïve Estimate for the Number of Human Cells in the Body**

**Fig 1:**
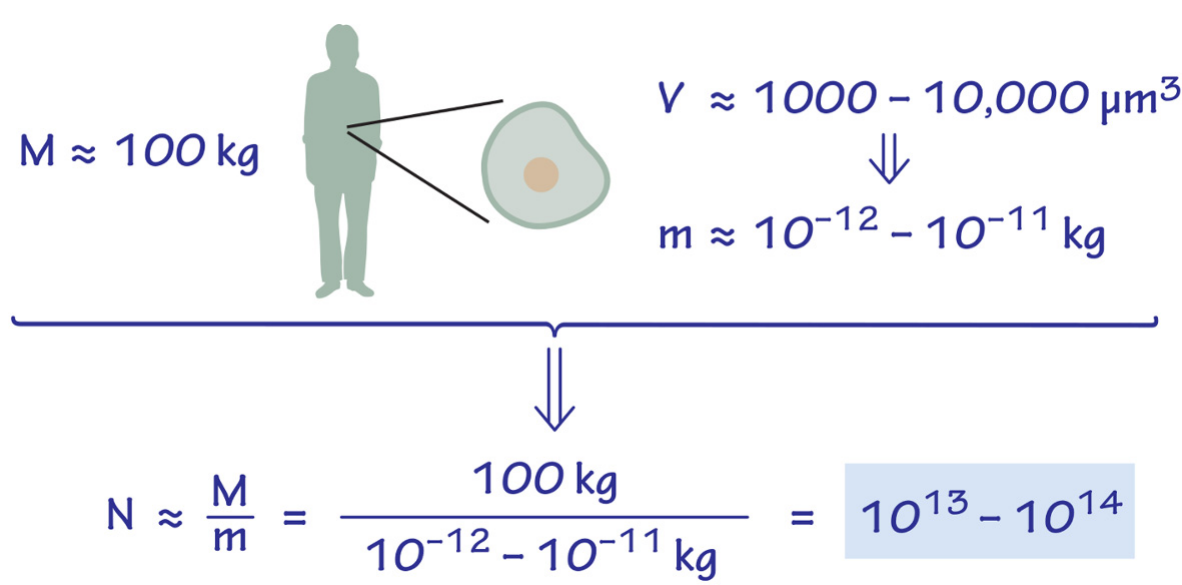
Back of the envelope estimate of the number of cells in an adult human body based on a characteristic volume and mass.

Assume a 10^2^ kg man, consisting of “representative” mammalian cells. Each mammalian cell, using a cell volume of 1,000–10,000 μm^3^_,_ and a cell density similar to that of water, will weigh 10^−12^–10^−11^ kg. We thus arrive at 10^13^–10^14^ human cells in total in the body, as shown in Fig 1. For these kind of estimates, where cell mass is estimated to within an order of magnitude, factors contributing to less than 2-fold difference are neglected. Thus, we use 100 kg as the mass of a reference man instead of 70 kg and similarly ignore the contribution of extracellular mass to the total mass. These simplifications are useful in making the estimate concise and transparent.

An alternative method that does not require considering a representative "average" cell systematically counts cells by type. Such an approach was taken in a recent detailed analysis [1]. The number of human cells in the body of each different category (by either cell type or organ system) was estimated. For each category, the cell count was obtained from a literature reference or by a calculation based on direct counts in histological cross sections. Summing over a total of 56 cell categories [1] resulted in an overall estimate of 3.7·10^13^ human cells in the body (SD 0.8·10^13^, i.e., CV of 22%).

### Updated Inventory of Human Cells in the Body

In our effort to revisit the measurements cited, we employed an approach that tries to combine the detailed, census approach with the benefits of a heuristic calculation used as a sanity check. We focused on the six cell types that were recently identified [1] to comprise 97% of the human cell count: red blood cells (accounting for 70%), glial cells (8%), endothelial cells (7%), dermal fibroblasts (5%), platelets (4%), and bone marrow cells (2%). The other 50 cell types account for the remaining 3%. In four cases (red blood cells, glial cells, endothelial cells, and dermal fibroblasts), we arrived at revised calculations as detailed in Box 4.

**Box 4. Revised Estimates for the Number of Red Blood Cells, Glial Cells, Endothelial Cells, and Dermal Fibroblasts**

The largest contributor to the overall number of human cells are red blood cells. Calculation of the number of red blood cells was made by taking an average blood volume of 4.9 L (SEM 1.6%, CV 9%) multiplied by a mean RBC count of 5.0·10^12^ cells/L (SEM 1.2%, CV 7%) (see Table 3 and S1 Data). The latter could be verified by looking at your routine complete blood count, normal values range from 4.6–6.1·10^12^ cells/L for males and 4.2–5.4·10^12^ cells/L for females. This led to a total of 2.5·10^13^ red blood cells (SEM 2%, CV 12%). This is similar to the earlier report of 2.6·10^13^ cells [1].

The number of glial cells was previously reported as 3·10^12^ [1]. This estimate is based on a 10:1 ratio between glial cells and neurons in the brain. This ratio of glia:neurons was held as a broadly accepted convention across the literature. However, a recent analysis [28] revisits this value and, after analyzing the variation across brain regions, concludes that the ratio is close to 1:1. The study concludes that there are 8.5·10^10^ glial cells (CV 11%) in the brain and a similar number of neurons and so we use these updated values here.

The number of endothelial cells in the body was earlier estimated at 2.5·10^12^ cells (CV 40%), based on the mean surface area of one endothelial cell [1] and the total surface area of the blood vessels, based on a total capillary length of 8·10^9^ cm. We could not find a primary source for the total length of the capillary bed and thus decided to revisit this estimate. We used data regarding the percentage of the blood volume in each type of blood vessels [29]. Using mean diameters for different blood vessels [30], we were able to derive (S1 Data) the total length of each type of vessel (arteries, veins, capillaries, etc.) and its corresponding surface area. Dividing by the mean surface area of one endothelial cell [31], we derive a reduced total estimate of 6·10^11^ cells.

The number of dermal fibroblasts was previously estimated to be 1.85·10^12^ [1], based on multiplying the total surface area (SA) of the human body (1.85 m^2^ [32]) by the areal density of dermal fibroblasts [33]. We wished to incorporate the dermal thickness (d) into the calculation. Dermal thickness was directly measured at 17 locations throughout the body [34], with the mean of these measurements yielding 0.11±0.04 cm. The dermis is composed of two main layers: papillary dermis (about 10% of the dermis thickness) and reticular dermis (the other 90%) [35]. The fibroblast density is greater in the papillary dermis, with a reported areal density, σ_pap._ of 10^6^ cells/cm^2^ (with 100 μm thickness of papillary, giving 10^8^ cells/cm^3^) [33]. The fibroblast density in the middle of the dermis was reported to be about 3·10^6^ cells/cm^3^ [36], giving an areal density of σ_ret._ = 3·10^5^ cells/cm^2^. Combining these we find: N_der.fib._ = SA·(σ_pap._ + σ_ret_.) = 1.85·10^4^ cm^2^ (10^6^ + 3·10^5^) cells/cm^2^ = 2.6·10^10^ cells. After this 100-fold decrease in number, dermal fibroblasts are estimated to account for only ≈0.05% of the human cell count.

**Fig 2:**
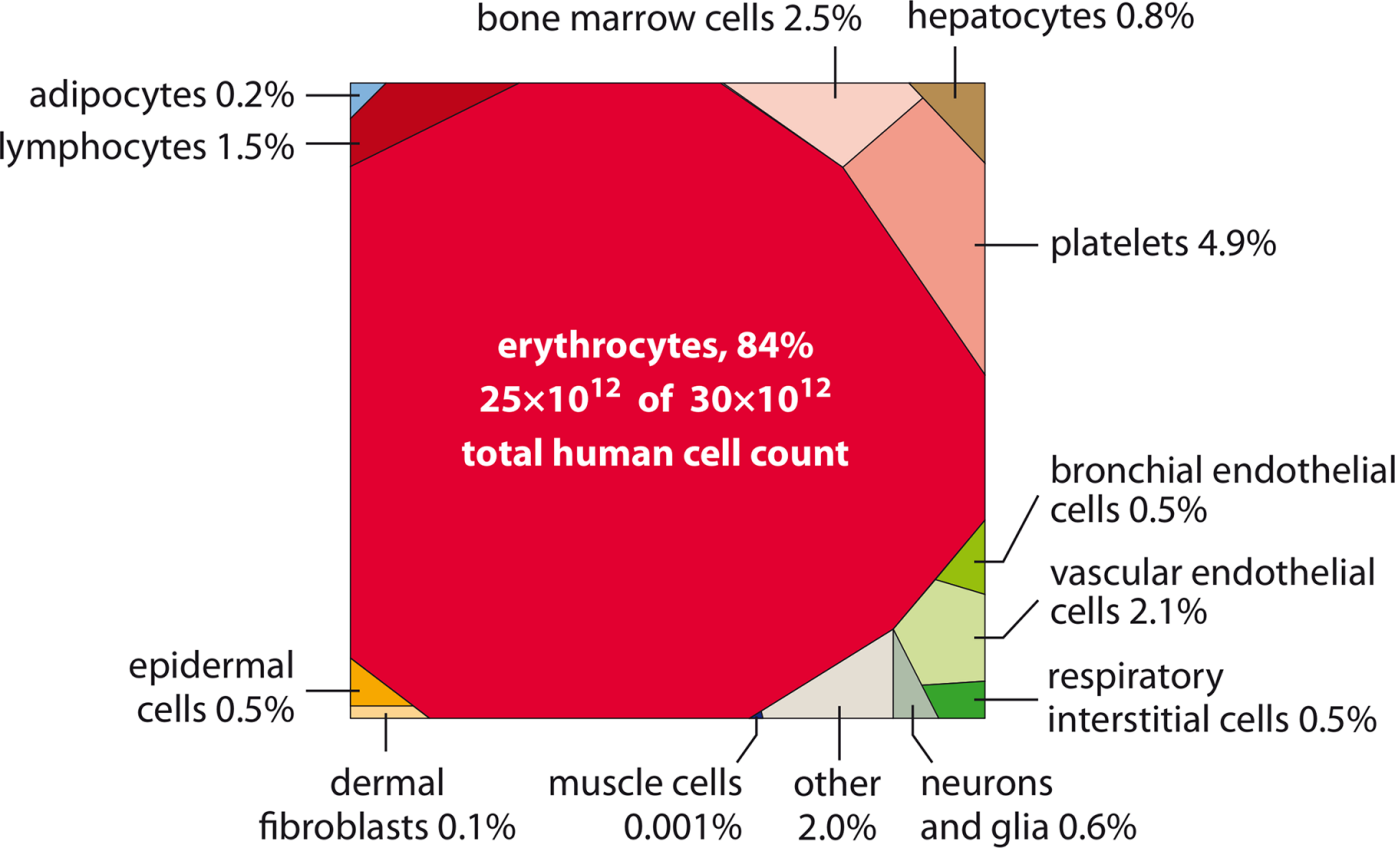
The distribution of the number of human cells by cell type. Representation as a Voronoi tree map where polygon area is proportional to the number of cells. Visualization performed using the online tool at http://bionic-vis.biologie.uni-greifswald.de/.

Our revised calculations of the number of glial cells, endothelial cells, and dermal fibroblast yield only 0.9·10^12^ cells, in contrast to 7.5·10^12^ cells in the previous estimate. This leaves us with 3.0·10^13^ human cells in the 70 kg “reference man,” with a calculated 2% uncertainty and 14% CV. We note that the uncertainty and CV estimates might be too optimistically low, as they are dominated by the reported high confidence of studies dealing with red blood cells but may underestimate systematic errors, omissions of some cell types, and similar factors that are hard to quantify. In Fig 2, we summarize the revised results for the contribution of the different cell types to the total number of human cells. Categories contributing >0.4% in cell count are presented. All the other categories sum up to about 2% together. We find that the body includes only 3·10^12^ non-blood human cells, merely 10% of the total updated human cell count. The visualization in Fig 2 highlights that almost 90% of the cells are estimated to be enucleated cells (26·10^12^ cells), mostly red blood cells and platelets, while the other ≈10% consist of ≈3·10^12^ nucleated cells. The striking dominance of the hematopoietic lineage in the cell count (90% of the total) is counterintuitive given the composition of the body by mass. This is the subject of the following analysis.

### Mass-Centered Approach as Sanity Check for Cell Count

It is prudent in making such estimates to approach the analysis from different angles. In that spirit, we now ask does the cumulative mass of the cells counted fall within the expected range for a reference adult? To properly tackle that question, we first need to state what the anticipated result is, i.e., total body cell mass. For a reference man mass of 70 kg, 25% is extracellular fluid [37], another 7% is extracellular solids [37], thus we need to account for ≈46 kg of cell mass (including fat).

**Fig 3:**
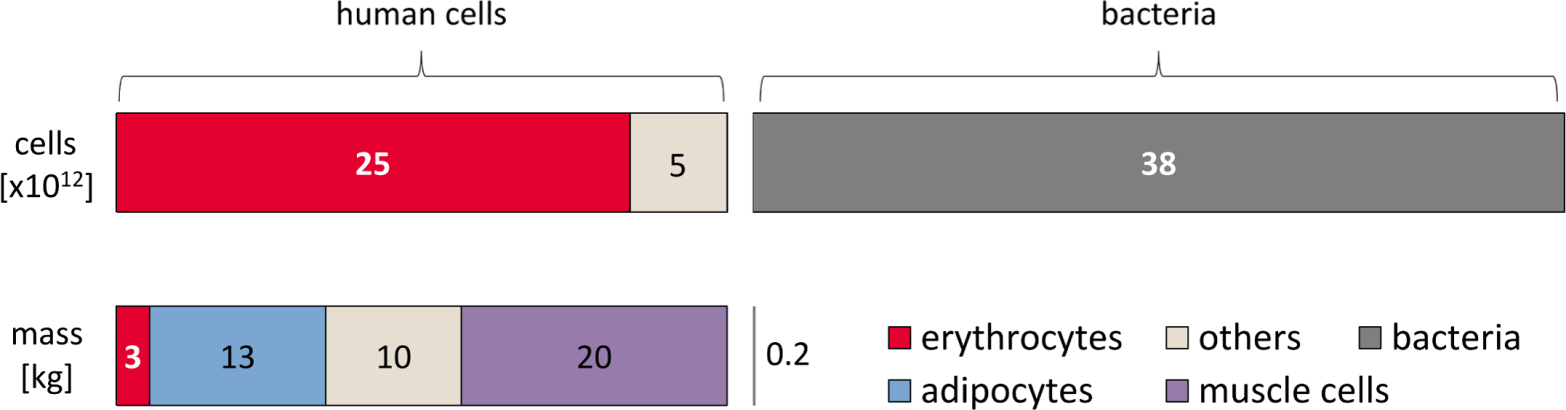
Distribution of cell number and mass for different cell types in the human body (for a 70 kg adult man). The upper bar displays the number of cells, while the lower bar displays the contribution from each of the main cell types comprising the overall cellular body mass (not including extracellular mass that adds another ≈24 kg). For comparison, the contribution of bacteria is shown on the right, amounting to only 0.2 kg, which is about 0.3% of the body weight.

A comprehensive systematic source for the composition of total cell mass (rather than total cell count) is the Report of the Task Group on Reference Man [6], which gives values for the mass of the main tissues of the human body. This mass per tissue analysis includes both intra- and extracellular components. To distinguish between intra- and extracellular portions of each tissue, we can leverage total body potassium measurements [38] as detailed in S1 Appendix. Fig 3 compares the main tissues that contribute to the human body, in terms of cell number and masses.

A striking outcome of this juxtaposition is the evident discordance between contributors to total cell mass and to cell number. The cell count is dominated by red blood cells (84%), among the smallest cell types in the human body with a volume of about 100 μm^3^. In contrast, 75% of total cell mass is composed of two cell types, fat cells (adipocytes) and muscle cells (myocytes), both large cells (usually >10,000 μm^3^ by volume) that constitute only a minute fraction (≈0.2%) of total cell number. At the other extreme, bacteria have a minor contribution in terms of mass, but a cell count comparable to all human cells combined, as discussed above. The mass balance accounts well for all expected body mass, giving support to our analysis. The option of overlooking a collection of very small cells numerous enough to alter the total cell count is further discussed in the S1 Appendix.

### The Ratio of Bacteria to Human Cells in the Adult Body

With the revised estimates for the number of human (3.0∙10^13^) and bacterial cells (3.8∙10^13^) in the body (the numerator and denominator of the B/H ratio), we can give an updated estimate of B/H = 1.3, with an uncertainty of 25% and a variation of 53% over the population of standard 70 kg males. This B/H value of about 1:1 (with the associated uncertainty range) should replace the 10:1 or 100:1 values that are stated in the literature until more accurate measurements become available.

We note that if one chooses to compare the number of bacteria in the human body (3.8·10^13^) to the number of nucleated human cells (≈0.3·10^13^), the ratio will be about 10:1. This is because the dominant population of non-nucleated red blood cells is not included in the calculation. We note that this ratio is the result of both the number of bacteria and the number of nucleated human cells in the body being several times lower than in the original 1970s estimate (that did not restrict the analysis to nucleated cells). The issue of whether cells without a nucleus should be included or discarded in the calculation of the number of human cells, and thus the B/H ratio, seems to be a question of definition. We view red blood cells as bona fide cells, as their name suggests. But it is also plausible to choose not to include them as some may think of them as “bags full of hemoglobin.” Inclusion of platelets in the count, which corresponds to their inclusion in previous counts, is also potentially disputable but has only a minor quantitative effect. Indeed, this opens an interesting tangential discussion on what should be defined as a cell.

### Variations in the Ratio of Bacteria to Human Cells across Population Segments

After reviewing the B/H ratio for the “reference man,” we now generalize our results by addressing other segments of the population. Looking at our estimate, we identify four main parameters that dominate the calculation:

1. colon volume
2. bacterial density in the colon
3. blood volume
4. hematocrit (i.e., red blood cells per unit volume).

These are the governing parameters due to the dominating contribution of the colonic bacteria and RBC count to the total bacterial and human cell counts, respectively. In order to evaluate the effect of gender, age, and obesity on the B/H ratio, we examine the change in these parameters across those groups.

Table 3 collects the changes to each of the previously mentioned parameters for individuals that represent different segments of the human population: reference adult woman (1.63 m, 60 kg [39]), young infant (age 4 weeks), infant (age 1 year), elder (66 years), and obese (140 kg).

Review of the literature shows no significant effect on the colonic bacterial concentrations over age from the one month old infant to the elderly [40,41]. The colonization of the neonatal GI tract from negligible colon bacterial concentrations of ≤10^5^ bacteria/mL to concentrations equivalent to those of adults occurs in just under one month [42]. For this dynamic period that is yet to be charted in high resolution, we do not supply a B/H ratio estimate. As with age, extremes of weight have low impact on bacterial cell counts. [43]. The reported values for infants and obese are in the range of variation of “the reference man.” In addition, we could not find any report in the literature on gender-specific differences in bacteria density in the colon. As can be appreciated from Table 3, the B/H ratio varies by up to 2-fold across those different population groups from a low of 1.3 to a high of 2.3.

**Table 3:** B/H ratio for different population. See Table B in S1 Appendix for full references.

population segment	body weight [kg]	age [y]	blood volume [L]	RBC count [10^12^/L]	colon content [g]	bac. conc. [10^11^/g wet] ^(1)^	total human cells [10^12^] ^(2)^	total bacteria [10^12^]	B:H
ref. man	70	20–30	4.9	5.0	420	0.92	30	38	**1.3**
ref. woman	63		3.9	4.5	480	0.92	21	44	**2.2**
young infant	4.4	4 weeks	0.4	3.8	48	0.92	1.9	4.4	**2.3**
infant	9.6	1	0.8	4.5	80	0.92	4	7	**1.7**
elder	70	66	3.8 ^(3)^	4.8	420	0.92	22	38	**1.8**
obese	140		6.7	5.0^(4)^	610^(5)^	0.92	40	56	**1.4**

^(1)^ No significant change in bacteria concentrations in relation to high variation for the reference man [40,43].

^(2)^ Assuming RBCs account for 84% of the total host cells as observed for the reference man.

^(3)^ Decrease of 24% in the blood volume, according to [44].

^(4)^ No significant change in the hematocrit in obesity [45].

^(5)^ We could not find any direct measurements of the colonic volume for obese individuals in the literature, yet from an indirect analysis the volume increases with weight and plateaus at about 600 mL [46].

We note that additional factors such as race and ethnicity may influence the B:H ratio. It has been shown that the bacterial population in the colon is strongly affected by geography [47], but current data is not enough to allow robust inference for the colonic concentrations and represents a data gap.

## Discussion

In this study, beyond providing up-to-date estimates on the average values of the number of cells, we aimed to give representative uncertainty ranges and the variation across population segments. This is based on comparing independent studies and the variation observed within studies.

The biggest knowledge gap we find is how realistic is the usage of the measured fecal bacteria density to represent also the average bacteria density in the colon. There is an inevitable gradient in bacteria concentration along the colon itself, from the low concentrations transiting from the ileum to the cecum of about 10^8^ bacteria/g to ~10^11^ bacteria/g measured in stool. The change in bacteria concentration arises from several factors, including water absorption that concentrates the bacteria in the colon, as well as from bacteria growth during the 1–2 day transit time and the shedding of bacteria from the mucosal surface. In some respects, the estimate we performed of multiplying observed fecal bacteria density with colon content volume can be considered an upper limit. More information on the relation between the actual densities of bacteria throughout the colon and those densities measured in feces will be a big step forward in improving the estimates of this study. Another element of uncertainty is the limited information on the volume of the colon content across individuals and conditions. These knowledge gaps indicate that there might be systematic errors beyond what we could account for in the uncertainty ranges we report.

In analyzing various population segments, our paper is clearly limited in scope. We touched on the obese, neonate, and elderly as well as the effect of gender but have not dealt with many other segments of interest, such as individuals in the course of antibiotic treatment or bowel preparation for colonoscopy, people with infections, chronic diseases of the GI tract, etc.

While we analyzed cell numbers, many researchers are interested in the number of genes as a reflection, for example, of the diversity of the microbiome metabolic capabilities. In order to properly estimate by what factor the genes in the bacteria we harbor outnumber our own twenty thousand genes, the very delicate question of what should be considered different genes must be properly defined, which is beyond the scope of this study.

We note in passing that the number of endosymbiotic bacteria that we harbor in the form of mitochondria probably outnumbers the body bacteria several fold. This can be appreciated by noting that most cell types (though not red blood cells) contain hundreds (or more) of mitochondria per cell [48].

Should we care about the absolute number of human cells in the body or the ratio of bacterial to human cells? Updating the ratio of bacteria to human cells from 10:1 or 100:1 to closer to 1:1 does not take away from the biological importance of the microbiota. Yet, we are convinced that a number widely stated should be based on the best available data, serving to keep the quantitative biological discourse rigorous. The study of absolute numbers is also of relevance for specific biological questions. For example, a recent study showed how knowing the number of cells in different tissues can be an important indicator in understanding variation in cancer risk among tissues [49]. Other applications refer to the dynamic processes of development and mutation accumulation. Finally, the type of numeric focus exercised here reveals and attracts attention to knowledge gaps such as the bacterial population densities in the proximal colon and how well are they represented by current analysis methods. We thus became aware through this study of promising steps forward in fulfilling the Delphic maxim of ‘‘know thyself” from a quantitative perspective.

## Supporting Information

S1 Appendix Supplementary information text. Elaboration of calculation methods and sanity check.
(DOCX)

S1 Data Detailed calculations. Spreadsheets with the detailed calculations mentioned throughout the text and references for all data sources.
(XLSX)

## Abbreviations

CV: coefficient of variation
FISH: fluorescent in situ hybridization
SD: standard deviation
SA: surface area
SEM: Standard Error of the Mean
