# Supplementary material for: Revised Estimates for the Number of Human and Bacteria Cells in the Body
Source: PLoS Biol. 2016 Aug 19;14(8):e1002533. doi: 10.1371/journal.pbio.1002533 (PMC4991899; doi:10.1371/journal.pbio.1002533)
Supplement: S1 Appendix — Elaboration of calculation methods and sanity check. (DOCX) [file pbio.1002533.s001.docx]

# Supplementary Information

# Revised estimates for the number of human and bacteria cells in the body

Ron Sender, Shai Fuchs & Ron Milo

# Contents

[1. Guide to detailed calculations in supplementary spreadsheet 1](#_Toc448340000)

[2. Mean bacterium mass in the colon 3](#_Toc448340001)

[3. An alternative method to derive the number of cells in the host based on DNA content 4](#_Toc448340002)

[4. Calculation of intra-cellular portions of tissues using potassium concentrations 4](#_Toc448340003)

[5. Analysis of potentially unaccounted cells using a mass-centered approach 5](#_Toc448340004)

[6. Detailed references for comparison of colonic bacterial concentrations 6](#_Toc448340005)

[7. Detailed references for estimations of B:H ratio for the general population 7](#_Toc448340006)

[8. Calculation of means, uncertainties and variation across the population 8](#_Toc448340007)

### 1. Guide to detailed calculations in supplementary spreadsheet

The S1 Data spreadsheet includes several tables that give details for the resources collected and calculations made throughout this study. Each tab provides calculations regarding one subject. Sheets are divided to several groups, as follows:

B:H Summary tabs:

- TotalBacteria - Derivation of total number of bacteria in the colon and the B/H ratio.
- BacterialConc - Summary of bacterial concentration in stool samples from the literature.
- ColonVolume - Derivation of colon volume value from the literature.
- B2HRatio - Calculation of the B/H ratio between the total bacteria number and the number of human cells.

Sanity check for the colon content:

- DailyFecal - Calculation of the mean daily fecal output from three literature sources.
- CTT - Derivation of colonic transit time (CTT) from [1].

Derivation of average bacterium mass from [2] is given in AvBacteriaSize tab.

Human cells count summary, integrating past calculations [3] and ours:

- HumanCellsSummary - Summary of the number of human cells in the body by tissue.
- HumanNucCellsSummary - Summary of the number of human nucleated cells in the body by tissue.
- TissueMass - Summary of cell mass and cell numbers in the main tissues.

Derivation of total RBC number, using blood volume and RBC counts:

- RBCcount - Calculation of the mean RBC count.
- BloodVol - Derivation of blood volume in the reference man according to four sources.
- RBC - Calculation of the number of red blood cells in the human body.

Revised calculation for several key cell types:

- DermalFib - Calculation of the number of dermal fibroblasts in the human body.
- Endothl - Calculation of the number of endothelial cells in the human body.

Variation in the ratio of bacteria to human cells across population segments:

- PopulationSegment - Summary of the effects of gender, age and obesity on the B:H ratio.
- GenderEffect - The effect of gender on the B:H ratio.
- AgeEffect - The effect of age on the B:H ratio (for infants and elderly).
- Obesity - The effect of obesity on the B:H ratio.

Additional tabs detailing past estimates for the number of human cells:

- Bianconi, 2013 - Summary of cells number and cell mass by tissues based on [3].
- HumanCell#ByDNA - Details of the estimate of human cells number from DNA content, described in [4].

### 2. Mean bacterium mass in the colon

What fraction of colonic content is occupied by bacterial mass? What is the mean mass of a bacterium in the colon? The measurements of bacterial concentrations in the colon (table 2), can be used to infer answers to those questions, provided two additional values: (1) the fraction of dried fecal mass that is dry bacteria and (2) the total water content in a bacterium. Overall dry mass fraction contributed by bacteria was directly measured to be 55% of fecal dry mass [2]. The dry mass percentage of cell mass varies for different types of bacteria [5,6] but can be assumed to be roughly equal to that of stool (29%), and thus the fraction of bacterial dry mass in dry feces is a good approximation to the fraction of bacterial mass in stool. Using the measured value of 4·10^11^ bacteria per gram dry stool [2], we evaluate the average mass of bacteria in the Stephen and Cummings samples to be 4.6·10^-12^g (SEM 35%, CV 47%). Interestingly, this value for the average bacterial cell mass is several times higher than is usually taken for a model bacterium such as *E. coli* [7,8].

###

### 3. An alternative method to derive the number of cells in the host based on DNA content

One study estimating the number of host cells used a DNA-centered method [4]. It estimated the number of cells in the body of a 25g mouse by dividing the total amount of DNA (stated to be 20 mg) by the DNA content of one diploid mouse cell (6·10^-12^ g DNA per cell) to get ≈3·10^9^ cells in a 25 g mouse. Then extrapolate from mouse to human by using the ratio of masses to get ≈10^13^ cells in the human body. This method excludes cells that do not contain DNA, such as red blood cells and platelets.

### 4. Calculation of intra-cellular portions of tissues using potassium concentrations

To distinguish between intra- and extra-cellular portions of each tissue we can leverage total body potassium measurements. The concentration of potassium in the intracellular and extracellular volumes of the body is known to be relatively constant [65]. Given these constant values, Wang [9,10] derived a formula connecting the potassium level of a tissue with its non-fat cell mass. The extracellular potassium concentration is only about 3% of the intracellular concentration and thus can be neglected to give the relation M_tissue_(kg)=0.0092 (kg/mmol) x [K] (mmol). We used this relation to derive the cell mass in each of the main tissues from reported potassium concentrations [6].

### 5. Analysis of potentially unaccounted cells using a mass-centered approach

Using the results cumulative mass of the cells counted (showed in Fig.3), we can check whether we have overlooked a collection of small cells that can change our results. If there exists a bulk of cells numerous enough to alter the total cell count it should contain on the order of 10^12^ cells or more. However, these cells cannot have a total mass more than a few kg at the most, as the mass of the reference body is already almost fully accounted for. Therefore, any such cells would need to have rather small mass, with an upper bound of 1 kg/10^12^ cells <1,000 pg/cell. Thus we conclude that if indeed there is any underestimation or omission in the cell count, for it to have a sizeable effect on total count, it should be of small cells.

The hematopoietic lineage has generally small cells and is a good candidate for undercounting. Bianconi et al [3] accounted most of the cells of this lineage: red blood cells, white blood cells and platelets were counted in the blood and in the bone marrow, but there is a non-negligible fraction of the white blood cells that presented outside of these tissues. The total number of lymphocytes in a 70 kg man is estimated to be about 5·10^11^ [11], most of them reside in the lymphatic system and in tissues across the body. Thus, while Bianconi et al. [3] have not considered lymphocytes outside the blood and bone marrow in their account, this underestimated population has only a marginal effect on the total cell count.

### 6. Detailed references for comparison of colonic bacterial concentrations

Table 2 of the manuscript gathers bacteria density measured in 14 literature sources. Here we provide full references for the mentioned articles.

*Table A: Values of bacteria density in stool as collected from literature sources.*

| **Article** | | | **bac. #/g dry stool (x10^11^)** | **dry matter as % of stool** | **bac. #/g wet stool (x10^11^)** | **CV(%)** |
| --- | --- | --- | --- | --- | --- | --- |
| **Author** | **Year** | **Ref.** |  |  |  |  |
| Houte & Gibbons | 1966 | [12] | **-** | **-** | **3.2** | 53% |
| Moore & Holdeman | 1974 | [13] | **5** | **22%** | **1.1** | 78% |
| Holdeman, Good & Moore | 1976 | [14] | **4.1** | **31%** | *1.3* | 66% |
| Stephen & Cummings | 1980 | [2] | **4** | 29%^(1)^ | *1.2* | 25% |
| Langendijk et al. | 1995 | [15] | **-** | **-** | **2.7** | 26% |
| Franks et al. | 1998 | [16] | **2.9** | **-** | *0.74*^(2)^ | 39% |
| Simmering & Kleessen | 1999 | [17] | **4.8** | **-** | *1.3*^(2)^ | 44% |
| Tannock et al. | 2000 | [18] | **-** | **-** | **0.95** | 40% |
| Harmsen, Raangs, He, Degener & Welling | 2002 | [19] | **2.1** | *30%* | **0.62** | 38% |
| Zoetendal et al | 2002 | [20] | **2.9** | **-** | *0.77*^(2)^ | 24% |
| Zhong et al. | 2004 | [21] | **1.5** | **23%** | *0.35* | 73% |
| Thiel & Blaut | 2005 | [22] | **3.5** | *25%* | **0.87** | 53% |
| He et al. | 2008 | [23] | **1.5** | **-** | *0.39*^(2)^ | 43% |
| Uyeno, Sekiguchi & Kamagata | 2008 | [24] | **-** | **-** | **0.44** | 34% |
| **Mean** |  |  | **-** | **27%±2%** | **0.92±19%** | **46%** |

*Mean bacteria number is calculated using the geometric mean to give robustness towards outlier values. Values quoted directly from the articles are written in bold, values derived by us are written in italic. Values reported with more than 2 significant digits are rounded to two significant digits as the uncertainty makes such over specification non sensible. ± standard error of the mean.*

1. *Value for* [2] *derived from their table 1.*
2. *From derivation, assuming the averaged dry matter fraction of 27%.*

### 7. Detailed references for estimations of B:H ratio for the general population

Table 3 of the manuscript reports best estimates for the B:H ratio in different segments of the population. Table B gives detailed references for these estimates.

*Table B: B/H ratio for different population segments*. See text for details.

| **Population**  **segment** | **body weight [kg]** | **age [y]** | **blood volume [L]** | **RBC count [10^12^/L]** | **colon content [g]** | **bac. conc. [10^11^/g wet]** ^(1)^ | **total human cells [10^12^]** ^(2)^ | **total bacteria [10^12^]** | **B:H** |
| --- | --- | --- | --- | --- | --- | --- | --- | --- | --- |
| [ref. man](file:///C:\Users\Ron\Dropbox\ביולוגיה\Human%20Cells\Writing\Submissions\bioRxiv\Detailed%20Calculations%20-%20160123RS.xlsx#B2HRatio!A1) | 70 | 20-30 | 4.9^(3)^ | 5.0^(4)^ | 420 | 0.92 | 30 | 38 | **1.3** |
| ref. [woman](file:///C:\Users\Ron\Dropbox\ביולוגיה\Human%20Cells\Writing\Submissions\bioRxiv\Detailed%20Calculations%20-%20160123RS.xlsx#GenderEffect!A1) | 63 |  | 3.9^(3)^ | 4.5^(4)^ | 480^(5)^ | 0.92 | 21 | 44 | **2.2** |
| [young](file:///C:\Users\Ron\Dropbox\ביולוגיה\Human%20Cells\Writing\Submissions\bioRxiv\Detailed%20Calculations%20-%20160123RS.xlsx#AgeEffect!A1) infant | 4.4^(6)^ | 4 weeks | 0.4^(7)^ | 3.8^(8)^ | 48 ^(9)^ | 0.92 | 1.9 | 4.4 | **2.3** |
| [infant](file:///C:\Users\Ron\Dropbox\ביולוגיה\Human%20Cells\Writing\Submissions\bioRxiv\Detailed%20Calculations%20-%20160123RS.xlsx#AgeEffect!A1) | 9.6^(6)^ | 1 | 0.8^(7)^ | 4.5^(8)^ | 80^(9)^ | 0.92 | 4 | 7 | **1.7** |
| [elder](file:///C:\Users\Ron\Dropbox\ביולוגיה\Human%20Cells\Writing\Submissions\bioRxiv\Detailed%20Calculations%20-%20160123RS.xlsx#AgeEffect!A1) | 70 | 66 | 3.8 ^(10)^ | 4.8^(11)^ | 420 | 0.92 | 22 | 38 | **1.8** |
| obese | 140 |  | 6.7^(12)^ | 5.0^(13)^ | 610^(14)^ | 0.92 | 40 | 56 | **1.4** |

1. No significant change in bacteria concentrations, in relation to high variation for the reference man [25,26].
2. Assuming RBCs account for 84% of the total host cells as observed for the reference man.
3. [27–30],^(4)^ [31–36], ^(5)^[37,38], ^(6)^[39], ^(7)^[40–42], ^(8)^[41,43],^(9)^[38].

^(10)^ Decrease of 24% in blood volume according to [44], ^(11)^[45], ^(12)^[29].

1. No significant change in the hematocrit in obesity [46].
2. We could not find any direct measurements of the colonic volume for obese individuals in the literature yet from an indirect analysis the volume increases with weight and plateaus at about 600 ml [47].

8. Calculation of means, uncertainties and variation across the population

Average values were calculated using the arithmetic mean of reported values except for bacterial concentration in the colon as described below. Uncertainty in our estimates is calculated as the standard error of the mean (SEM), calculated as the standard deviation of mean values, divided by the square root of the number of reported values. For the calculation of variation across the population, the coefficient of variation (CV) was calculated as the arithmetic mean of the ratio between the standard deviation and the mean, of each of the reported values.

For the derivation of the bacterial concentration in the colon content, a large range of values was gathered from 14 different articles. The estimate for representative average value was calculated in two ways: the geometric mean and the median of the reported results. In the second case SEM was calculated from the empirical distribution using bootstrapping by the standard deviation of 1000 repeats (see S1 Data tab BacterialConc.).

#### Bibliography

[1] Southwell BR, Clarke MCC, Sutcliffe J, Hutson JM. Colonic transit studies: normal values for adults and children with comparison of radiological and scintigraphic methods. Pediatr Surg Int 2009;25:559–72. doi:10.1007/s00383-009-2387-x.

[2] Stephen A, Cummings J. The microbial contribution to human faecal mass. J Med Microbiol 1980;13:45–56.

[3] Bianconi E, Piovesan A, Facchin F, Beraudi A, Casadei R, Frabetti F, et al. An estimation of the number of cells in the human body. Ann Hum Biol 2013;40:463–71. doi:10.3109/03014460.2013.807878.

[4] Baserga R. The Biology of Cell Reproduction. Harvard University Press; 1985.

[5] Robertson BR, Button DK. Determination of the Biomasses of Small Bacteria at Low Concentrations in a Mixture of Species with Forward Light Scatter Measurements by Flow Cytometry. Appl Environ Microbiol 1998;64:3900–9.

[6] Bratbak G, Dundas I. Bacterial dry matter content and biomass estimations. Appl Environ Microbiol 1984;48:755–7.

[7] Chesbro W, Evans T, Eifert R. Very Slow Growth of Escherichia coli 1979;139:625–38.

[8] Kubitschek HE, Friske JA, Counter-analyzer C, Trueba FS, England N, Instruments B. Determination of Bacterial Cell Volume with the Coulter Counter 1986;168:1466–7.

[9] Wang Z, Heshka S, Heymsfield SB, Shen W, Gallagher D. A cellular-level approach to predicting resting energy expenditure across the adult years. Am J Clin Nutr 2005;81:799–806.

[10] Wang Z, St-Onge M-P, Lecumberri B, Pi-Sunyer FX, Heshka S, Wang J, et al. Body cell mass: model development and validation at the cellular level of body composition. Am J Physiol Endocrinol Metab 2004;286:E123–8. doi:10.1152/ajpendo.00227.2003.

[11] Trepel F. Number and distribution of lymphocytes in man. A critical analysis. Klin Wochenschr 1974;52:511–5.

[12] Houte JVAN, Gibbons RJ. Studies of the cultivable flora of normal human feces 1966;32:212–22.

[13] Moore WE, Holdeman L V. Human fecal flora: the normal flora of 20 Japanese-Hawaiians. Appl Microbiol 1974;27:961–79.

[14] Holdeman L V, Good IJ, Moore WE. Human fecal flora: variation in bacterial composition within individuals and a possible effect of emotional stress. Appl Environ Microbiol 1976;31:359–75.

[15] Langendijk PS, Schut F, Jansen GJ, Raangs GC, Kamphuis GERR, Wilkinson MHF, et al. Quantitative Fluorescence In Situ Hybridization of Bifidobacterium spp . with Genus-Specific 16S rRNA-Targeted Probes and Its Application in Fecal Samples. Appl Environ Microbiol 1995;61:3069–75.

[16] Franks AH, Harmsen HJM, Gerwin C, Jansen GJ, Schut F, Gjalt W. Variations of Bacterial Populations in Human Feces Measured by Fluorescent In Situ Hybridization with Group-Specific 16S rRNA-Targeted Oligonucleotide Probes. Appl Environ Microbiol 1998.

[17] Simmering R, Kleessen B. Quantification of the Flavonoid-Degrading Bacterium Eubacterium ramulus in Human Fecal Samples with Species-Specific Oligonucleotide Hybridization Probe. Appl Environ Microbiol 1999;65:3705–9.

[18] Tannock GW, Munro K, Harmsen HJM, Welling GW, Smart J, Gopal PK. Analysis of the Fecal Microflora of Human Subjects Consuming a Probiotic Product Containing Lactobacillus rhamnosus DR20. Appl Environ Microbiol 2000;66:2578–88. doi:10.1128/AEM.66.6.2578-2588.2000.

[19] Harmsen HJM, Raangs GC, He T, Degener JE, Welling GW. Extensive Set of 16S rRNA-Based Probes for Detection of Bacteria in Human Feces. Appl Environ Microbiol 2002;68:2982–90. doi:10.1128/AEM.68.6.2982.

[20] Zoetendal EG, Ben-Amor K, Harmsen HJM, Schut F, Akkermans ADL, De Vos WM. Quantification of Uncultured Ruminococcus obeum -Like Bacteria in Human Fecal Samples by Fluorescent In Situ Hybridization and Flow Cytometry Using 16S rRNA-Targeted Probes Quantification of Uncultured Ruminococcus obeum -Like Bacteria in Human Fecal Samp. Appl Environ Microbiol 2002;68:4225–32. doi:10.1128/AEM.68.9.4225.

[21] Zhong Y, Priebe MG, Vonk RJ, Huang C-Y, Antoine J-M, He T, et al. The Role of Colonic Microbiota in Lactose Intolerance. Dig Dis Sci 2004;49:78–83. doi:10.1023/B:DDAS.0000011606.96795.40.

[22] Thiel R, Blaut M. An improved method for the automated enumeration of fluorescently labelled bacteria in human faeces. J Microbiol Methods 2005;61:369–79. doi:10.1016/j.mimet.2004.12.014.

[23] He T, Priebe MG, Zhong Y, Huang C, Harmsen HJM, Raangs GC, et al. Effects of yogurt and bifidobacteria supplementation on the colonic microbiota in lactose-intolerant subjects. J Appl Microbiol 2008;104:595–604. doi:10.1111/j.1365-2672.2007.03579.x.

[24] Uyeno Y, Sekiguchi Y, Kamagata Y. Impact of consumption of probiotic lactobacilli-containing yogurt on microbial composition in human feces. Int J Food Microbiol 2008;122:16–22. doi:10.1016/j.ijfoodmicro.2007.11.042.

[25] Roger LC, Mccartney AL. Longitudinal investigation of the faecal microbiota of healthy full-term infants using fluorescence in situ hybridization and denaturing gradient gel electrophoresis. Microbiology 2010:3317–28. doi:10.1099/mic.0.041913-0.

[26] Vulevic J, Juric A, Tzortzis G, Gibson GR. A Mixture of trans -Galactooligosaccharides Reduces Markers of Metabolic Syndrome and Modulates the Fecal Microbiota and Immune Function of Overweight Adults 1 – 3. J Nutr 2013:324–31. doi:10.3945/jn.112.166132.galactooligosaccharides.

[27] Boer P. Estimated lean body mass as an index for normalization of body fluid volumes in humans. Am J Physiol 1984;007:632–6.

[28] Snyder WS, Cook MJ, Nasset ES, Karhausen LR, Parry Howells G, Tipton IH. Report of the Task Group on Reference Man. vol. 23. Pergamon Press: Oxford; 1975. doi:10.1016/S0074-2740(75)80015-8.

[29] Feldschuh J, Enson Y. Prediction of the normal blood volume. Relation of blood volume to body habitus. Circulation 1977;56:605–12. doi:10.1161/01.CIR.56.4.605.

[30] Nadler SB, Hidalgo JH, Bloch T. Prediction of blood volume in normal human adults. Surgery 1962;51:224–32.

[31] Wakeman L, Al-Ismail S, Benton A, Beddall A, Gibbs A, Hartnell S, et al. Robust, routine haematology reference ranges for healthy adults. Int J Lab Hematol 2007;29:279–83. doi:10.1111/j.1365-2257.2006.00883.x.

[32] Nordin G, Mårtensson a, Swolin B, Sandberg S, Christensen NJ, Thorsteinsson V, et al. A multicentre study of reference intervals for haemoglobin, basic blood cell counts and erythrocyte indices in the adult population of the Nordic countries. Scand J Clin Lab Invest 2004;64:385–98. doi:10.1080/00365510410002797.

[33] Pekelharing JM, Hauss O, Jonge R De, Lokhoff J, Sodikromo J, Spaans M, et al. Haematology reference intervals for established and novel parameters in healthy adults. Diagnostic Perspect 2010;1:1–11.

[34] Ambayya A, Su AT, Osman NH, Nik-Samsudin NR, Khalid K, Chang KM, et al. Haematological reference intervals in a multiethnic population. PLoS One 2014;9:1–7. doi:10.1371/journal.pone.0091968.

[35] Dosoo DK, Kayan K, Adu-Gyasi D, Kwara E, Ocran J, Osei-Kwakye K, et al. Haematological and biochemical reference values for healthy adults in the Middle Belt of Ghana. PLoS One 2012;7:1–9. doi:10.1371/journal.pone.0036308.

[36] Volkmer B, Heinemann M. Condition-dependent cell volume and concentration of Escherichia coli to facilitate data conversion for systems biology modeling. PLoS One 2011;6:e23126. doi:10.1371/journal.pone.0023126.

[37] Pritchard SE, Marciani L, Garsed KC, Hoad CL, Thongborisute W, Roberts E, et al. Fasting and postprandial volumes of the undisturbed colon: normal values and changes in diarrhea-predominant irritable bowel syndrome measured using serial MRI. Neurogastroenterol Motil 2013;26:124–30. doi:10.1111/nmo.12243.

[38] ICRP. Basic anatomical and physiological data for use in radiological protection: reference values. ICRP Publication 89. vol. 32. Pergamon; 2002. doi:10.1016/S0146-6453(03)00002-2.

[39] World Health Organization. WHO Child Growth Standards based on length/height, weight and age. Acta Paediatr. Suppl., vol. 450, Geneva: World Health Organization; 2006, p. 76–85. doi:10.1080/08035320500495548.

[40] Howie SR. Blood sample volumes in child health research : review of safe limits. Bull World Heal Organ 2011;89:46–53. doi:10.2471/BLT.10.080010.

[41] Matoth Y, Zaizov R, Varsano I. Postnatal Changes in Some Red Cell Parameters. Acta Paediatr 1971;60:317–23.

[42] Russell SJM. Blood Volume Studies in Healthy Children. Arch Dis Child 1949;24:88–98.

[43] Zierk J, Arzideh F, Rechenauer T, Haeckel R, Rascher W, Metzler M, et al. Age- and Sex-Specific Dynamics in 22 Hematologic and Biochemical Analytes from Birth to Adolescence. Clin Chem 2015;61:964–73. doi:10.1373/clinchem.2015.239731.

[44] Davy KP, Seals DR. Total blood volume in healthy young and older men. Am Physiol Soc 1994;i:2059–62.

[45] Adeli K, Raizman JE, Chen Y, Higgins V, Nieuwesteeg M, Abdelhaleem M, et al. Complex Biological Profile of Hematologic Markers across Pediatric , Adult , and Geriatric Ages : Establishment of Robust Pediatric and Adult Reference Intervals on the Basis of the Canadian Health Measures Survey: Clin Chem 2015;61:1075–86. doi:10.1373/clinchem.2015.240531.

[46] Retzlaff JA, Tauxe WN, Kiel JM, Stroebel CF. Erythrocyte Volume, Plasma Volume, and Lean Body Mass in Adult Men and Women. Blood 1969;33:649–67.

[47] Young JF, Luecke RH, Pearce BA, Lee T, Ahn H, Baek S, et al. Human Organ / Tissue Growth Algorithms that Include Obese Individuals and Black / White Population Organ Weight Similarities from Autopsy Data. J Toxicol Environ Health 2009;72:527–40. doi:10.1080/15287390802647203.
